# Recent advances in light patterned optogenetic photostimulation in freely moving mice

**DOI:** 10.1117/1.NPh.11.S1.S11508

**Published:** 2024-02-23

**Authors:** Antonio Lorca-Cámara, François G. C. Blot, Nicolò Accanto

**Affiliations:** Sorbonne Université, INSERM, CNRS, Institut de la Vision, Paris, France

**Keywords:** fiber-optic applications, optogenetics, multiphoton microscopy, holography applications, functional imaging, neurophotonics

## Abstract

Optogenetics opened the door to a new era of neuroscience. New optical developments are under way to enable high-resolution neuronal activity imaging and selective photostimulation of neuronal ensembles in freely moving animals. These advancements could allow researchers to interrogate, with cellular precision, functionally relevant neuronal circuits in the framework of naturalistic brain activity. We provide an overview of the current state-of-the-art of imaging and photostimulation in freely moving rodents and present a road map for future optical and engineering developments toward miniaturized microscopes that could reach beyond the currently existing systems.

## 1 Introduction

Understanding the connections between neuronal activity and behavior stands as a fundamental goal in neuroscience that requires the precise mapping and/or manipulation of neuronal activity. Genetically encoded calcium indicators^1^^,^^2^ and voltage indicators^3^^,^^4^ have made it possible to image neuronal activity. Concurrently, the emergence of optogenetics,^5^^–^^7^ based on light-gated ion channels (opsins), has provided the means to optically manipulate neurons. On the optical side, advances in multiphoton microscopy^8^^,^^9^ have provided tools to image neuronal activity with cellular resolution, deeper into the tissue (>1  mm^10^^,^^11^), with fast acquisition rates,^12^^,^^13^ and on ultra-large (up to 5 mm^14^^,^^15^) fields of view (FOVs). Simultaneously, progress in wavefront-shaping techniques, such as computer-generated holography (CGH)^16^ using liquid crystal spatial light modulators (SLMs), coupled with high-energy ultrafast lasers, have unlocked the precise manipulation of groups of neurons, down to the single-cell level.^17^^–^^20^ The combination of these approaches has enabled cellular resolution *in vivo* imaging and manipulation studies, often referred to as all-optical studies,^21^^–^^26^ which enabled identification of functionally relevant neuronal ensembles, replaying and/or altering their spatiotemporal activity profile, and deciphering their behavioral implications. Importantly, the selective control of even a reduced number (<20) of functionally defined neurons showed significant impact on the behavioral output.^22^^,^^26^

Nevertheless, these advanced optical methods were primarily designed for benchtop microscopes and typically necessitate using head restraints on animals under an objective. Head fixation can alter perception and interaction with the environment, interfering with sensory integration and motor output, and it induces stress in the animal, leading to biased neuronal integration.^27^ Head restriction has been showed to affect not only motor-related neuronal circuits but also a number of networks related to cognitive functions, such as the recruitment and coding of hippocampal place cells during navigation,^28^ or the multisensory encoding of V1 neurons for visual flow integration.^29^ All together these studies question our ability to reproduce neuronal coding resulting from voluntary real-world exploration, in artificial/virtual settings.^30^^,^^31^ Although the lack of vestibular and head/neck proprioception inputs have been emphasized to explain the differences in neuronal activity between virtual reality systems and real-world exploration, a larger range of senses could be involved (smell and hearing), raising the idea that active free motion is a behavioral state in essence,^32^ comparable to sleep or other known awake states (drowsy, alert, and resting). There is thus a need for tools to observe and manipulate neuronal circuits with high resolution in freely moving animals to investigate how natural behaviors shape neuronal processing in the brain.

To this end, miniaturized optical systems have been developed to image neuronal activity during natural behaviors. Three main families of systems are used today.

**Fig. 1:**
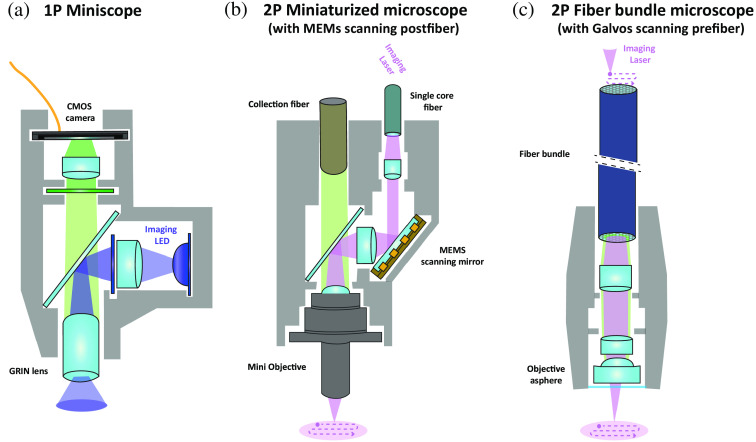
Optical systems for neuronal activity imaging in freely moving mice. Schemes of the optical elements and the light paths of: (a) a 1P miniscope, (b) a 2P miniaturized microscope, and (c) a 2P fiber bundle-based microscope, and the illumination on the imaging plane, wide field (a) or scanning (b) and (c) MEMS, miniature electromagnetic mirrors; CMOS, complementary metal-oxide-semiconductor; and GRIN lens, gradient refracting-index lens. Detailed information of the three systems is presented in Table 1.

• *1P Miniscopes*.^33^^,^^34^ One photon (1P) head-mounted wide-field miniscopes use a LED source, microlenses, and a miniature CMOS camera to image neuronal activity [Fig. 1(a)]. While these devices enable functional imaging of large FOVs^35^^–^^37^ at high acquisition rates^38^ within a cost-effective system, they suffer from poor optical sectioning, and suboptimal signal-to-noise ratio (SNR) due to the out-of-focus fluorescence background.
• *Miniaturized multiphoton microscopes*.^39^^–^^43^ They are based on single-core optical fibers that propagate infrared light from a pulsed laser to the animal head to generate two-photon (2P)^44^^–^^46^ or three-photon (3P)^47^^,^^48^ excitation. A light and miniaturized scanner, based either on microelectromechanical system (MEMS^49^) scanning mirrors that deflect the laser beam or on a fiber scanning unit that moves the fiber tip in a spiral trajectory^45^ [Fig. 1(b)], quickly scans the diffraction limited laser beam on the sample to generate an image. If these systems offer the highest optical resolution and penetration depth compared to other miniaturized optical systems, the single-core fiber delivery has so far remained incompatible with different imaging techniques, such as random access microscopy^12^^,^^50^ or multipoint scanless excitation.^51^
• *Fiber bundle-based microscopes*. In this case, a multicore fiber, also called fiber bundle, composed of many thousands of individual cores, is used to relay a standard optical system to the animal’s head [Fig. 1(c)]. Fiber bundle microscopes have been demonstrated in freely moving animals both in the 1P^52^^–^^54^ and 2P^55^^–^^57^ regime. Thanks to the multicore delivery, the use of fiber bundles is in general compatible with other imaging techniques, such as multipoint excitation,^52^ structured illumination,^52^^,^^54^ or multipoint confocal imaging.^53^ At the same time, the intercore space limits the optical resolution and lowers the light transmission, especially for the excitation laser.

**Table 1:** Comparison of the main imaging parameters of recently published 1P miniscopes, 2P/3P miniaturized microscopes, and fiber bundle-based microscopes.

System	Main components	FOV and resolution	Frame rate	Achievable depth	3D possible?	Advantages/limitations
1P miniscopes	LED, miniaturized camera	FOV $4.8\times 3.6\;{\mathrm{mm}}^{2}$ Lat. res. 5 μm (Ref. 36) FOV $8\times 10\;{\mathrm{mm}}^{2}$ Lat. Res. 50 μm (Ref. 37)	500 Hz (Ref. 38)	Superficial layers ∼100 μm	With light-field techniques (Refs. 58 and 59)	Fast acquisition, large FOV, wireless possibilities/1P resolution and depth limitations
2P miniaturized microscopes	Pulsed laser, single-core fiber, MEMS	FOV $420\times 420\;{\mu m}^{2}$ Lat. Res. 1.2 μm Axial res. 18 μm (Ref. 44) FOV $1\times 0.8\;{\mathrm{mm}}^{2}$ Lat. res. 1.47 μm Axial res. 25 μm (Ref. 46)	40 Hz (Ref. 44)	<400 μm	With tunable lenses (Refs. 44 and 46)	High resolution in deeper regions/ Not shown with different imaging or photostimulation techniques
3P miniaturized microscopes	Pulsed laser, single-core fiber, MEMS	FOV $400\times 400\;{\mu m}^{2}$ Lat. res. ∼1 μm Axial res. ∼10 μm (Refs. 47 and 48)	∼15 Hz (Refs. 47 and 48)	<1.2 mm	With tunable lenses (Ref. 48)	Access to deeper regions/lower frame rate
1P fiber bundle microscopes	Visible lasers, benchtop scanners/DMD	FOV ∼250 μm-wide Lat. res. 2 μm Axial res. 10 to 40 μm (Refs. 52–54)	200 Hz (Ref. 53)	260 μm*in vitro* 120 μm*in vivo* with structured illumination (Ref. 54)	With tunable lenses (Ref. 54)	Compatible with optimized illumination and photostimulation/1P resolution and depth limitations
2P fiber bundle microscopes	Pulsed lasers, benchtop scanners/SLM	FOV ∼250 μm-wide Lat. res. 2 μm Axial res. 10 to 15 μm (Refs. 56 and 57)	50 Hz (Ref. 57)	∼150 μm*in vivo* (Refs. 56 and 57)	With tunable lenses. (Ref. 56)	Compatible with optimized illumination and photostimulation/optical resolution limited by core size

Unfortunately, most existing systems are currently only compatible with imaging of neuronal activity and cannot perform optogenetic photostimulation with single-cell spatial resolution, in freely moving animals. The ability to also photostimulate neuronal ensembles in freely moving animal holds major insights to correlate microcircuits control with behavioral outputs. In the following sections, we review the few existing systems to deliver optogenetic photostimulation to the brain in freely moving mice, with a particular attention to systems that can provide near single-cell resolution photostimulation, and explore potential future developments to overcome current limitations.

## 2 Current Optical Systems for Optogenetic Photostimulation in Freely Moving Mice

### 2.1 Optoelectronics for Optogenetic Photostimulation

Optogenetics, in its simplest form, employs an optical fiber to deliver wide-field 1P illumination, therefore already compatible with the study of freely moving animals.^60^^,^^61^ Various techniques were subsequently developed to increase the spatial precision of light delivery and/or to deliver light at multiple points in the brain.^62^ Among those, implantable microLED arrays^63^^–^^66^ provide reprogrammable illumination patterns at the millisecond scale for optogenetic control in the brain of freely moving animals. Alternatively, multiple fibers (up to several tens) were implanted at different brain regions and separately addressed for both fiber photometry and optogenetic photostimulation.^67^ Finally, tapered optical fibers^68^^–^^70^ or photonics waveguides^71^ also allow some control over the depth at which light is emitted via mode or wavelength-division multiplexing. However, none of these devices are compatible with simultaneous imaging and photostimulation with single-neuron resolution, which is of great importance to understand how neuronal circuits encode information.

### 2.2 Systems for All Optical Studies in Freely Moving Animals

Only few innovative systems have emerged for near single-cell resolution imaging and optogenetic photostimulation in freely moving animals. They are mainly based either on subsequent developments of the 1P miniscope architecture, or on the use of fiber bundles.

#### 2.2.1 1P Miniscopes for wide-field imaging and photostimulation

Miniscopes can readily be combined with cable-connected LED probes for optogenetic stimulation of brain regions distal from the imaging FOV.^72^ Integrating the optoelectronic circuit into the miniscope offers precise synchronization of optogenetic manipulation with imaging recording. This greatly facilitates accurate *post hoc* trace analysis and enables multisites optogenetic stimulation with a single imaging FOV, providing insights into long-range connectivity *in vivo*. Alternatively, systems with two LED sources at different wavelength bands^73^ or different lasers^74^ were developed to enable imaging and photostimulation over the same FOV. However, these systems are limited to wide-field illumination for photostimulation, which does not enable the investigation of refined microcircuits. An interesting future perspective could be to couple microLED arrays from the previous section with a 1P miniscope to provide higher resolution and reconfigurable patterned photostimulation on one brain region, with the simultaneous 1P calcium imaging on a different region.

#### 2.2.2 1P Miniscope and 1P fiber bundle microscopes for patterned illumination

**Fig. 2:**
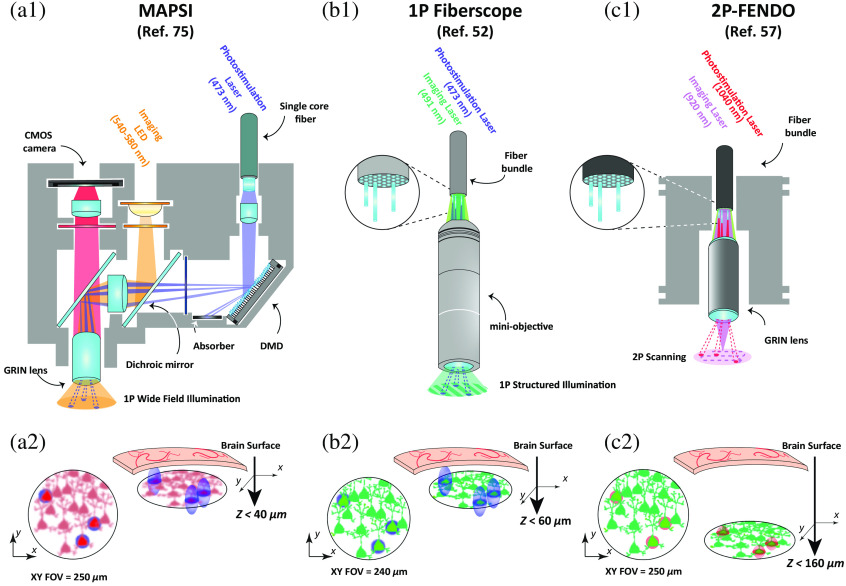
All-optical systems for patterned illumination in freely moving animals. (a1) 1P MAPSI^75^ system using widefield imaging with an LED and patterned photostimulation with a DMD within a FOV of 250  μm-diameter. The fluorescence (in red, as the calcium indicator jRCaMP1b was used in the experiment) is detected with a miniaturized CMOS camera. (a2) Single-cell resolution photostimulation was proven down to 40  μm below the GRIN lens surface. (b1) 1P fiberscope^52^ that propagates two visible wavelength lasers for imaging and photostimulation from a standard benchtop microscope to the brain using a fiber bundle and a mini-objective. (b2) The FOV for the imaging (using the green calcium indicator GCaMP5-G) and the holographic photostimulation is 240  μm-diameter. Single-cell resolution photostimulation was proven down to 60  μm deep. (c1) 2P fiberscope (2P-FENDO)^57^ using a fiber bundle and a GRIN lens to transmit the 2P excitation for both the imaging (using the green calcium indicator jGCaMP7s) and holographic photostimulation from the benchtop microscope to the head the mice. (c2) The FOV is 250  μm-diameter. Single-cell resolution photostimulation was proven down to 160  μm below the GRIN lens surface. (a2)–(c2) Representations of the x/y view of the imaged cells [red (a2) or green (b2)/(c2)] in the FOV (left) with the photostimulation spots [1P excitation blue spots in (a2)/(b2) and 2P excitation red spots in (c2)], and the x/y/z view (right) to illustrate the axial extension of the photostimulation spots (better axial resolution is obtained in c2 when using 2P excitation), together with the maximal reachable depth from the brain surface (largest in c2 for 2P excitation). The imaging quality is qualitatively illustrated with higher or lower blurring applied to the FOV and is lower for 1P widefield imaging (a2) and higher for 1P imaging with structured illumination (b2), and 2P imaging (c2) DMD, digital micromirror device; CMOS, complementary metal-oxide-semiconductor; and GRIN lens, gradient refracting-index lens. Detailed information of the three systems is presented in Table 2.

1P miniscopes can be enhanced by incorporating a miniaturized DMD for spatial light patterning in freely moving animals, as demonstrated in the miniscope with all-optical patterned stimulation and imaging (MAPSI) system^75^ [Fig. 2(a)]. Using a collimated laser beam, MAPSI ensures lateral resolutions of ∼10  μm and an axial resolution of 30 to 40  μm, on a 250  μm wide FOV, sufficient to achieve near single-neuron stimulation in freely moving animals. However, as a consequence of the 1P illumination and the scattering of the brain, the penetration depth at which near single-cell resolution photostimulation was achieved remained limited to the first ∼50  μm below the gradient refractive index (GRIN) lens used.^75^ Additionally, while conventional miniscopes typically weight <5  g, the MAPSI system weights 7.8 g (25% to 30% of the animal weight), which necessitates the use of a weight carrier.

**Table 2:** Comparison of the main imaging and photostimulation parameters of recently published μLED systems, 1P miniscopes, and fiber bundle-based microscopes.

System	Illumination mode	FOV and resolution stim.	Limit depth	Multiplane	Advantages/limitations
μLED	Multiple μLEDs for array illumination	FOV limited by the size of the array Cone of illumination	Implant, no theoretical limitations	Yes, along the shaft	Implantable in deep regions + wireless possibilities/no cellular resolution and no flexibility to target user-desired neurons
1P MAPSI (Ref. 75)	1P laser + single-core fiber, DMD for patterned illumination	FOV 250 μm-diameter Lat. res. 10 μm Axial res. 30 μm	<40 μm	No	Miniaturized optics/lack of cellular resolution in depth and weight (7.8 g)
1P fiber bundle microscope (Ref. 52)	1P laser + fiber bundle, SLM for patterned illumination	FOV 250 μm-diameter Lat. res. ∼5 μm Axial res. ∼18 μm	<60 μm	No	Light weight/1P resolution and depth limitations
2P-FENDO (Ref. 57)	2P laser + fiber bundle, SLM for patterned illumination	FOV 250 μm-diameter Lat. res. ∼10 μm Axial res. ∼10 μm	<160 μm	No	Light weight, 2P resolution and depth access/no multiplane, lateral resolution limited by core size

An alternative strategy is to use optical fiber bundles to simultaneously transmit the imaging source and the patterned photostimulation as well as to collect the fluorescence from calcium indicators, as shown in Ref. 52 for the first time [Fig. 2(b)]. Such a system offered, on a 240  μm wide FOV, an experimentally defined axial resolution of 18 for 5  μm large photostimulation spots, sufficient to achieve near single-cell resolution photostimulation. However, as for the MAPSI system, near single-cell photostimulation was only possible within <60  μm deep from the brain surface. To improve penetration depth and spatial resolution of both the imaging and the photostimulation spots and reduce background noise, multiphoton microscopy can be employed.

#### 2.2.3 2P All-optical studies with a fiber bundle

Recently, we have developed a two-photon fiberscope, 2P-FENDO,^57^ based on an optical fiber bundle, to both record and optogenetically manipulate neuronal populations with single-cell resolution in freely moving mice [Fig. 2(c)]. 2P-FENDO uses extended spots encompassing multiple fiber cores for both imaging and photostimulation, thereby reducing the power density and preventing self-phase modulation effects that can disrupt the excitation pulse.^76^ Importantly, we have demonstrated that the inherent intercore delays of a fiber bundle decompose the excitation spot in time, to ensure single-cell axial resolution (∼10  μm) and prevent out-of-focus excitation, even for extended illumination spots. With 2P-FENDO, we have achieved functional imaging at a frame rate of up to 20 Hz within a 2D FOV of 250  μm in diameter, together with high-resolution photostimulation of selected groups of neurons using an SLM to pattern the light entering the fiber bundle. 2P-FENDO demonstrated near single-cell photostimulation precision, as it only induced detectable calcium responses in neurons that were within 20  μm from the photostimulation spot (spot diameter of 10  μm). The 2P excitation regime allowed us to access deeper regions within the brain (depths of up to 160  μm) below the brain surface.

However, the limited size of the FOV and the lower optical resolution defined by the intercore spacing, together with the inhomogeneity of 2P excitation through different cores of the fiber bundle (characterized for different types of bundles in Ref. 77), result in lower imaging quality compared to the previously described multiphoton miniaturized microscopes.^44^^,^^45^^,^^47^

## 3 Perspectives for All-Optical Systems in Freely Moving Mice

The currently available all-optical systems developed for the study of freely moving mice all present advantages and disadvantages with respect to spatial resolution, diameter of the FOV, penetration depth, system complexity, flexibility, and weight. New efforts from the neurophotonics community will be necessary to improve these technologies to a level comparable to standard benchtop microscopes and ensure their widespread accessibility.

### 3.1 Micro-Optic Engineering

**Fig. 3:**
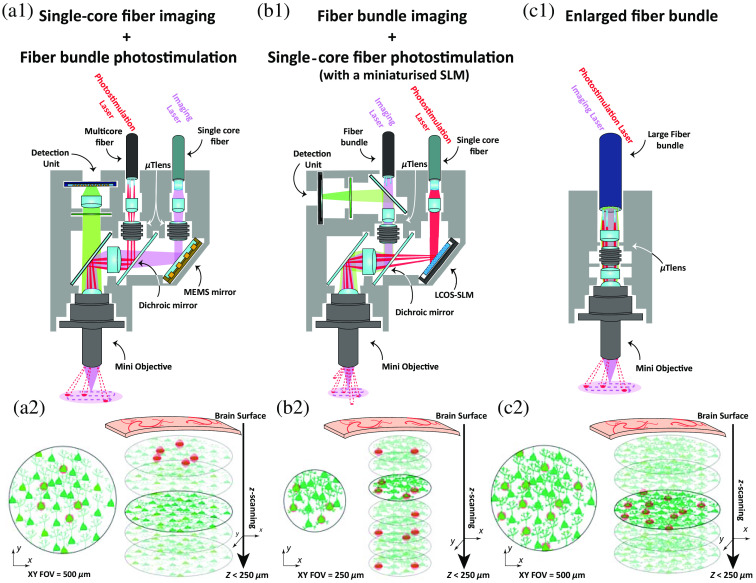
Possible all-optical architectures for patterned illumination in freely moving animals. (a1)–(c1) Schemes of the optical elements, the light paths, and the scanning on the imaging plane. (a2)–(c2) Representations of the x/y view of the imaged cells (green) in the FOV (left) with the photostimulation spots (red spots), and the x/y/z view (right) to illustrate the axial extension of the photostimulation spots, together with the expected reachable depth from the brain surface. The imaging quality is illustrated by a Gaussian blur applied on the FOV as we compare 2P imaging through a single-core fiber (a2), and 2P imaging through fiber bundles (b2/c2). Representations of the expected imaging quality (green) in the FOV (left) and the photostimulation spots (red dots), with the reachable depth from the brain surface (right), when using 2P excitation (larger depth could be achieved with 3P excitation^47^^,^^48^). The mini objective and tunable lens (μTlens) could be for instance the one presented in Ref. 44. (a2), (b2) We consider the FOV for imaging and photostimulation to be the largest ones so far demonstrated when using a single-core optical fiber and MEMS scanners (Ref. 44) and a fiber bundle (Refs. 56 and 57) in the 2P regime, while in c2 a larger FOV comes from the optimization of the fiber bundle and distal optics as explained in the text. (a2) Independent tunable lenses could enable the decoupling of the imaging and photostimulation planes. (b2) The miniaturized SLM at the distal end of the fiber would give access to 3D light multiplexing. (c2) A single tunable lens would shift simultaneously the imaging and photostimulation plane, but on a larger FOV. MEMS, microelectro-mechanical systems; μTlens, microtunable lens; and LCOS-SLM, liquid crystal on silicon SLMs.

One potential improvement is to integrate a multiphoton miniaturized microscope (such as MINI2P, Ref. 44) for the best image quality with a single-cell resolution patterned photostimulation system based on a fiber bundle, similar to 2P-FENDO^57^ [an example of such a system is depicted in Fig. 3(a)]. This will require substantial optical, mechanical, and electronic engineering efforts, especially given the critical need to minimize the weight on the animal’s head. The future availability of high-performance miniaturized optical components (both active and passive) will undoubtedly ease its implementation. Recent developments in high-resolution three-dimensional (3D) printing offer a promising route, allowing for the direct fabrication of aberration corrected and optimized microlenses on top of optical fibers,^78^^–^^80^ as well as GRIN lenses.^81^

### 3.2 Miniaturized Spatial Light Modulators

Targeting arbitrary three-dimensional distributions of cells at the sample plane is of great importance in optogenetic applications.^20^ However, this requires phase modulation (such as in CGH), which is challenging in freely moving animals as the phase information is mixed across different modes of a multimode fiber or different cores of a multicore fiber. Wavefront shaping strategies^82^ using an SLM before the fiber have been used to compensate for phase variations and refocus a beam without additional lenses at the fiber output,^83^^–^^87^ but remain highly sensitive to the fiber bending, which has so far prevented their application in freely moving animals, even if progress in this sense is underway.^85^^,^^88^ An alternative strategy to achieve 3D light targeting could be to use a miniaturized SLM at the fiber output, in a configuration similar to the MAPSI system.^75^ However, the compact DMD used in the MAPSI is highly inefficient when used as amplitude modulator and would require complex (and again inefficient) optical designs to be used as a phase modulator,^75^ hindering its application in the 2P regime. The development of a portable, lightweight phase-only SLM [as illustrated in Fig. 3(b)] that can be incorporated directly at the animal head would be disruptive for all-optical 2P fiberscopes and thus constitutes a promising direction for the neurophotonics field. Apart from miniaturizing existing liquid crystal SLM technology (starting for example from the LUNA-NIR-147 model from Holoeye), active and reconfigurable metasurfaces and matrices of tunable lenses could constitute a promising alternative that has undergone much progress in recent years.^89^^–^^91^

### 3.3 Fiber-Optic Engineering

All-optical systems based on fiber bundles offer the advantage of requiring minimal optics at the distal end of the fiber (2P-FENDO only uses a single GRIN lens after the fiber), which limits weight and obstruction. Major improvements in these systems^56^^,^^57^ will result from enhanced imaging quality, larger FOVs, and higher SNR. The image quality is affected by the inhomogeneities in 2P excitation,^77^ the core to core coupling,^92^ and the intercore distance of the fiber bundle ($d_{\text{core-core}}$), while the size of the FOV (${\mathrm{FOV}}_{\max}$) is determined by the diameter of the bundle ($\phi_{\text{fiber}}$) and the magnification of the optics at the distal end of the fiber (M). *Ad hoc* design of larger-in-diameter yet flexible bundles with a sufficiently small intercore distance ($d_{\text{core-core}}$) to maintain high lateral resolution ($d_{xy}$), $d_{xy}=d_{\text{core-core}}/M$, and a reduced 2P inhomogeneity, together with optimized distal optics,^46^ will increase the FOV (${\mathrm{FOV}}_{\max}=\phi_{\text{fiber}}/M$) and improve the image quality. Fiber engineering, therefore, presents a promising avenue to optimize all-optical studies in freely moving animals [as seen in Fig. 3(c)].

Finally, one effective way to improve the imaging SNR is using more complex scanning or multiplexing strategies, which are in general difficult to implement in a multiphoton miniaturized microscope. For instance, one could avoid scanning areas of the FOV that carry no information. This could be reached with random access microscopy^12^^,^^50^ or even with a scanless approach^51^ that uses CGH to excite only the cells of interest.

## 4 Concluding Remarks

In this article, we have reviewed the state-of-the-art for all-optical studies in freely moving mice and we have given different routes to optimize the performances of these devices to match standards of current benchtop microscopes. Miniaturized systems for all-optical studies will provide an important addition in the near future to understand how discrete neuronal networks shape behavior in animals that are free to move.

It is essential to highlight that a common challenge of all imaging devices working in freely moving animals is motion artifacts. Although movements in the recorded image can be compensated with motion correction postprocessing algorithms,^93^^,^^94^ achieving single-cell optogenetic targeting along the experiment would require online correction to compensate for potential motions of the FOV. Lateral displacements of the FOV could be compensated with a fast SLM, using a fast phase recalculation^95^ to adapt the stimulation pattern to the FOV movements and maintain single-cell resolution. All-optical studies of freely moving animals will therefore also largely benefit from further algorithm developments as well as computational imaging.

As a final remark, while optogenetics takes its very first steps in clinical applications,^96^ preclinical studies demonstrated the important role that patterned illumination will play in future therapeutic applications.^97^^–^^99^ Optical means to implement light delivery targeting are predicted to make important contribution for a novel class of brain–machine interfaces^100^ and to translate optogenetic neuronal control to the clinics. We believe that the concepts described in this article will help guiding further developments.

## Data Availability

Data sharing is not applicable to this article, as no new data were reported.
